# Ethanol Ablation as a Treatment in a Low-Risk Follicular Thyroid Cancer: A Case Report

**DOI:** 10.7759/cureus.27960

**Published:** 2022-08-13

**Authors:** Juan Pesantez, Carla Lituma, Carla Valencia, Jose Prieto, Marco Cazorla

**Affiliations:** 1 Internal Medicine, University of Cuenca, Cuenca, ECU; 2 Internal Medicine, California Institute of Behavioral Neurosciences & Psychology, Fairfield, USA; 3 Internal Medicine, Loyola University MacNeal Hospital, Berwyn, USA; 4 Endocrinology, Diabetes, and Metabolism, Jose Carrasco Arteaga Hospital, Ecuadorian Institute of Social Security, Cuenca, ECU

**Keywords:** thyroid nodules, ethanol ablation, papillary tumor, follicular tumor, thyroid cancer

## Abstract

Thyroid nodules are relatively prevalent in clinical practice; they are found by palpation in 3-7% of the general population and by ultrasonography (US) in roughly 50%. Image-guided nonsurgical procedures such as ethanol ablation (EA) or radiofrequency ablation (RFA) have been proposed for a selected group of patients as alternatives to traditional treatments. We present a case of a low-risk follicular variant of papillary thyroid cancer treated successfully with EA after examination and identification of the nodule as an ideal candidate for the treatment proposed. We highlight the efficacy of EA in this case, and how this contributes to the existing literature to continue proposing this as a viable treatment option.

## Introduction

Thyroid nodules are relatively prevalent in clinical practice; they are found by palpation in 3-7% of the general population and by ultrasonography (US) in roughly 50% [1]. Thyroid cancer incidence has increased in industrialized countries over the last 20 to 30 years, with mortality remaining stable or with minimal changes. Small papillary thyroid carcinomas (PTCs) account for the bulk of new thyroid cancer cases. These small, low-risk lesions are frequently discovered by chance in patients with no symptoms [2]. PTCs represent at least 80-85% of follicular cell-derived thyroid cancers [3].

Ethanol has been used therapeutically for decades in various indications such as an antiseptic, an antidote for methanol or ethylene glycol intoxication, alcohol withdrawal syndrome, and also as an ablating, sclerosing, or embolizing agent [4]. When ethanol is injected into cystic thyroid nodules, it promotes cellular dehydration and protein denaturation, resulting in reactive fibrosis [5]. In oncology, ethanol has proven its efficacy in treating various types of cancer, including hepatocellular carcinoma, renal cell carcinoma, and lately recurrent malignant thyroid cancers when the patients presented with high surgical risk or refused surgery [4].

The traditional management options of treating all patients with thyroid cancer with surgery, radioactive iodine (RAI), and suppressive thyroid hormone therapy are no longer appropriate. Thyroid cancer patients should be treated based on their overall prognosis, recurrence, mortality risks, therapy advantages, and hazards, as well as the patient's background and values. As a result, image-guided nonsurgical procedures such as ethanol ablation (EA) or radiofrequency ablation (RFA) have been proposed for a selected group of patients as alternatives to external radiation and surgical excision [1,2]. In terms of efficacy, side effects, cost, or quality of life, none of the indications for ethanol injection therapy has been adequately compared to alternative nonsurgical approaches. Therefore, EA is currently only suggested as a first-line treatment agent for benign recurring thyroid cysts [6]. Nonetheless, the usage of EA in localized, recurrent, PTCs, whether in the thyroid bed or regional lymph nodes, is becoming more common [6].

EA is usually a safe treatment for recurrent thyroid nodules, either benign or malignant. However, the patients could present with localized pain in the puncture site (usually the most common side effect), hematomas, facial flushing, drunken sense, dyspnea, temporary hyperthyroidism, and hoarseness, which is an important complication as a result of damage to the recurrent laryngeal nerve. Hoarseness regularly occurs when the target lesion is localized in the thyroid bed or around the carotid bed. Most cases last several days to weeks and completely recover within three to six months [1].

It is important to mention that despite the existing literature and proven outcomes of this treatment, it is very uncommon to use this in Ecuador. During this review, we have not found any existing literature on patients who underwent the mentioned treatment in this country. Thus, we consider it very important to contribute this information to the medical community in this country and help to improve the medical management of patients.

## Case presentation

Patient information

A 75-year-old female with a past medical history of hypertension and prediabetes presented to the endocrinologist's office for follow-up. Her surgical history was positive for hysterectomy and rotator cuff tear injury. She denied relevant family history and psychosocial or genetic diseases. At the moment of the assessment, she was asymptomatic.

Clinical findings

During the physical examination, an approximately 8-mm mass on the right side of the neck was noted. The mass was nonpainful and mobile, with a negative transillumination test and negative lymph node enlargement. General and systemic examination showed no alterations.

Diagnostic assessment

**Figure 1:**
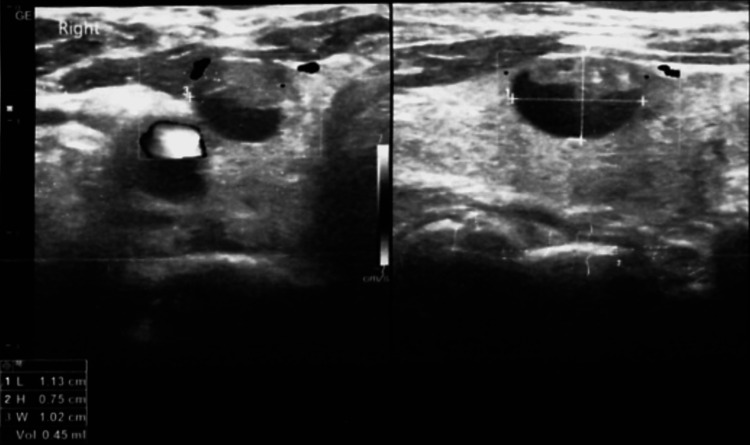
Neck ultrasound showing the thyroid nodule with cystic features and microcalcifications

The patient had a normal complete blood count, with thyroid-stimulating hormone (TSH) at 2.913 U/mL and free T4 at 1.2 ng/dl. A neck ultrasound revealed an 11 x 7 x 10 mm nodule on the thyroid's right lobe with central microcalcifications and regular borders (Figure 1). Additionally, a second nodule measuring 2 x 2 mm located near the first one with regular borders and no calcifications was found. No signs of lymph node enlargement or metastasis were seen. Under ultrasound guidance, fine needle aspiration was performed for the bigger nodule. Cytology results evidenced a normocellular extension composed of isolated polymorphonuclear leukocytes, scattered follicular cells, and others arranged in groups with changes compatible with an encapsulated follicular variant of papillary thyroid cancer.

Therapeutic intervention

**Figure 2:**
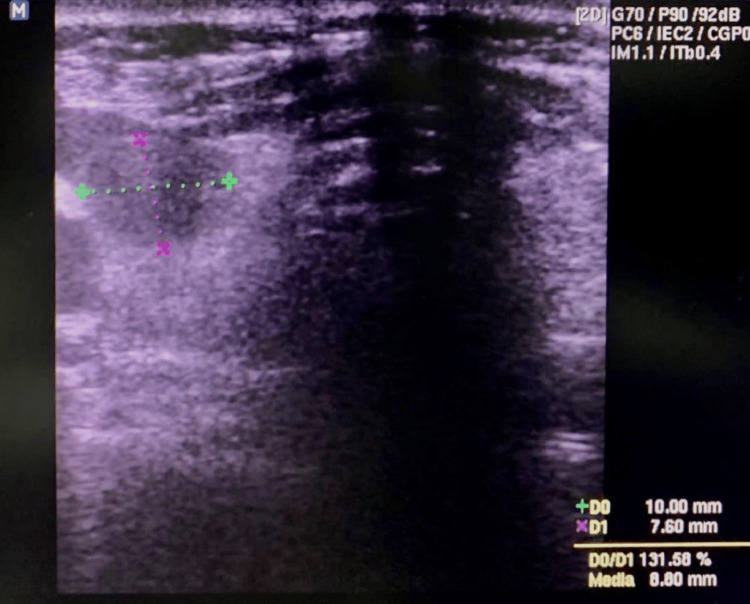
Neck ultrasound after aspiration of fluid from the cystic space

**Figure 3:**
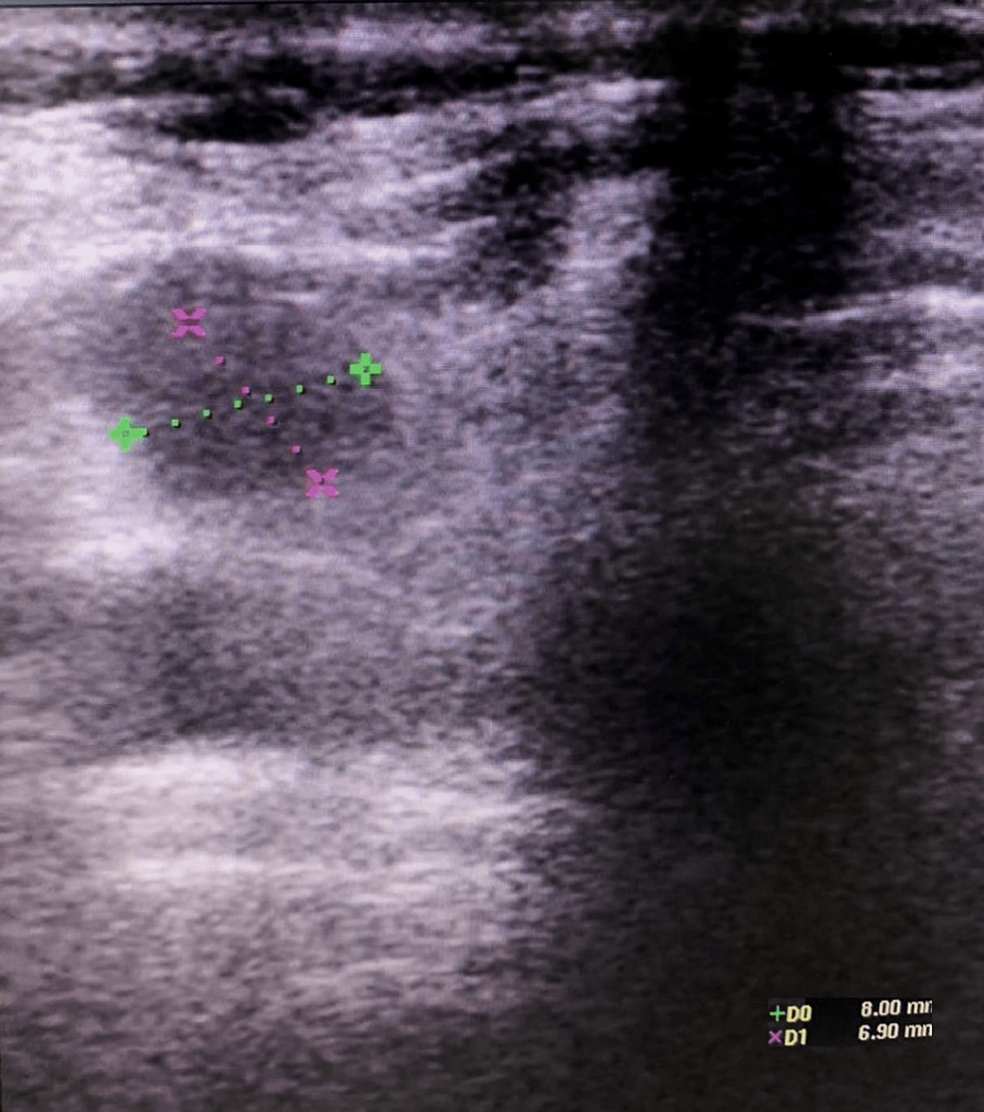
Reduction in volume of cystic space after ethanol injection

After skin sterilization, lidocaine at 2% was injected at the puncture site. Under ultrasound guidance, a 20 G needle was inserted into the center of the cystic area, and approximately 0.5 mm^3^ of fluid was obtained through aspiration with a syringe. The ultrasound showed a size reduction of the cyst (10 x 7 mm in diameter) (Figure 2). Subsequently, 3.5 mm^3^ of 99% ethanol was instilled in the previous aspirated cystic space, leaving the ethanol within the cyst (Figure 3). Once the process was finished, the needle was carefully removed, and the puncture site was lightly compressed for around five minutes without any complications.

Follow-up and outcomes

Once the procedure was finished, a new ultrasound was performed, showing a reduction in the size of the cyst (8 x 6 mm in diameter) (Figure 3). The patient complained about mild pain on the procedure site and a transient drunken sense. She was discharged after observation for one hour without complications, with ibuprofen to treat the pain and a follow-up schedule in five months.

**Figure 4:**
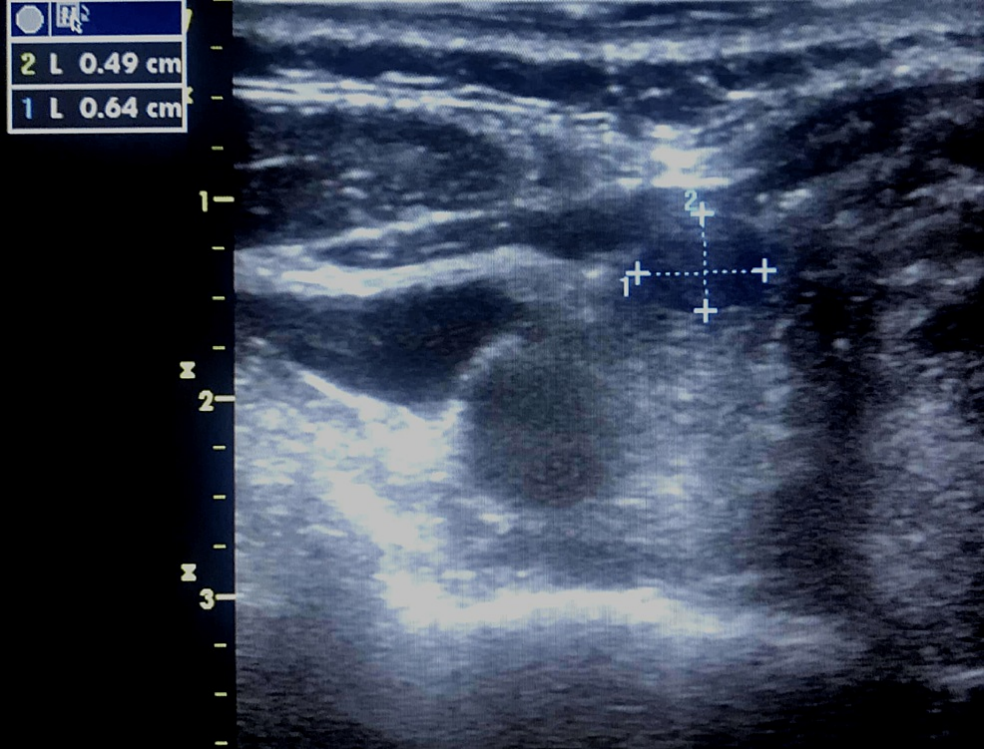
Neck ultrasound showing a 4 x 6 mm (0.49 x 0.64 cm) thyroid cystic mass, compatible with shrinkage of the previously treated thyroid nodule

At the five-month follow-up consultation, the patient was asymptomatic, and physical examination was unremarkable. Neck ultrasound revealed a 4 x 6 mm cystic thyroid mass, compatible with shrinkage of 43.3% for length and 34.6% for the height of the initial nodule size (Figure 4).

## Discussion

The vast majority of the literature describes the EA technique as a safe and inexpensive procedure for patients with benign thyroid nodules and recurrent papillary thyroid cancer. Tofé et al. described that in 42 patients with differentiated recurrent thyroid cancer, 71 lesions were found and treated with EA. Of those, 62 lesions (87.32%) demonstrated complete ablation after a mean follow-up of 40.5 months [7].

In an eight years study conducted by Monzani et al., 132 patients, aged 47.5 ± 12.9 years, diagnosed with an autonomous thyroid nodule, received percutaneous ethanol therapy. In all the patients, the intervention was able to obtain a considerable reduction in nodule volume. Recurrence of hyperthyroidism was never seen in the entirely healed individuals during their follow-up; however, five partially cured patients presented relapse [8]. Additionally, Tarantino et al. reported 125 patients diagnosed with hyperfunctioning thyroid nodules who underwent EA. Three of those patients refused to complete the EA therapy due to pain. The results showed that all 122 patients exhibited normal thyroid function tests. In 93% of them, absent uptake of iodine in the nodules and later recovery of normal uptake in the thyroid parenchyma were observed. Subsequent follow-up assessment revealed shrinkage of 112 of the ablated nodules in about 50-90% (mean 66%) of the original size [9].

Furthermore, this technique can also be applied as a minimally invasive therapy alternative for primary tumors in patients with papillary thyroid cancer who refuse to undergo more invasive procedures [2]. Hay et al. described 17 biopsy-proven tumor foci in 15 patients aged 36 to 86 years with adult papillary thyroid microcarcinoma (APTM), who were treated with EA and followed for a median of 64 months. There were no complications, all 17 tumors were reduced in size (median shrinkage of 93%) and Doppler flow was eliminated. Further follow-up showed that eight tumors (47%) were undetectable by ultrasound after a median of 10 months, and no new papillary thyroid microadenoma foci or nodal metastases were found on subsequent visits [3].

Ozderya et al. described that in 55 cystic and mixed property nodules treated with percutaneous ethanol injection, the procedure caused a cystic shrinkage of 80.7% at six months and 82.1% at 12 months of follow-up, underlining that the volume reduction was more evident in the purely cystic nodules than in the mixed ones. In addition, the smaller nodules had a greater reduction after the procedure in the 12th month. The patients showed no signs of serious complications [10].

In our patient, the tumor was a follicular variant of papillary thyroid cancer, the attending physician offered the different therapeutic approaches for this pathology (radioactive iodine ablation, surgical excision, and EA), explaining the risks and benefits of each one of them in detail. Due to the refusal of the patient to be subject to a surgical procedure and the low-risk characteristics of the tumor, in this particular case, i.e., encapsulated follicular variant of papillary thyroid cancer with no local or distant metastases, clinical N0, no tumor invasion of loco-regional and vascular tissues, no aggressive cytotype, and intra-thyroidal well-differentiated follicular thyroid cancer (Table 1), a less invasive procedure such as EA was performed.

**Table 1:** ATA 2009 risk stratification system with proposed modifications for structural disease recurrence in differentiated thyroid cancer Adapted from Iñiguez-Ariza et al. [2]. ATA, American Thyroid Association; DM, distant metastases; EFVPTC, encapsulated follicular variant of papillary thyroid cancer; ETE, extrathyroidal extension; FTC, follicular thyroid cancer; Gross ETE, macroscopic invasion of tumor into the perithyroidal soft tissues; LN, lymph nodes; N0, no evidence of regional lymph node metastases; N1, metastases to regional node; PMC, papillary microcarcinoma; PTC, papillary thyroid carcinoma; RAI, radioactive iodine; WBS, whole body scan; WDFTC, well-differentiated follicular thyroid cancer.

ATA low-risk	ATA intermediate risk	ATA high risk
PTC with all of the following:	Microscopic ETE	Gross ETE
No local or DM	RAI-avid metastatic foci in the neck on the first post-treatment WBS	Incomplete tumor resection
All macroscopic tumor has been resected	Aggressive cytotype (e.g., tall cell, hobnail variant, and columnar cell carcinoma)	Distant metastases
No tumor invasion of loco-regional tissues or structures	PTC with vascular invasion	Postoperative serum thyroglobulin suggestive of DM
No aggressive cytotype (e.g., tall cell, hobnail variant, and columnar cell carcinoma)	Clinical N1 or >5 pathologic N1 with all involved LN <3cm in the largest dimension	Pathologic N1 with any metastatic LN ≥3 cm in the largest dimension
If RAI was given, there are no RAI-avid metastatic foci outside the thyroid bed on the first post-treatment WBS	Multifocal PMC with ETE and BRAF^V600E^ mutated (if known)	FTC with extensive vascular invasion (>4 foci of vascular invasion)
No vascular invasion		
Clinical N0 or ≤5 pathologic N1 micro-metastases (<0.2 cm in largest dimension)		
Intra-thyroidal EFVPTC		
Intra-thyroidal WD-FTC with capsular invasion and no or minimal (<4 foci) vascular invasion		
Intra-thyroidal PMC, unifocal or multifocal including BRAF^V600E^ mutated (if known)		

## Conclusions

EA has shown promising results in treating thyroid nodules, especially benign ones. Nevertheless, as seen in many different studies, it has become more frequent to use this technique for low-risk malignant nodules as well. However, big-sample studies are needed to obtain more conclusive results. As stated in different case reports, the patient's satisfaction is evident, and a factor to consider when treating these conditions. Also, the few side effects and short-term recovery are some advantages that EA can provide over more invasive procedures. Thus, it is imperative for physicians to consider the option of EA for their patients as a viable and secure treatment option for a thyroid nodule, especially if they have factors that limit the usage of other options.
